# Solar Thrill: Using the Sun to Cool Vaccines

**DOI:** 10.1289/ehp.115-a208

**Published:** 2007-04

**Authors:** Adrian Burton

Using the sun to keep vaccines cool? It sounds like a contradiction in terms, but this is exactly the technology being developed by a partnership between UNEP OzonAction, UNICEF, the WHO, the Danish Technological Institute, Greenpeace, the German governmental group GTZ Proklima, the international nonprofit organization PATH, and the private sector companies Vestfrost and Danfoss. The SolarChill fridge requires no batteries or any other energy inputs to stay cool, so it emits no carbon dioxide. In addition, it requires little maintenance, is cheap to run, and uses no ozone-damaging refrigerants. It may sound too good to be true, but successful field tests have now set this technology on the road to WHO approval.

Many vaccines—for example, those consisting of attenuated, live viruses such as polio and measles vaccines—must be kept at temperatures of 2–8ºC to be effective. “This requires refrigeration, which in turn requires a power source that is one hundred percent reliable twenty-four hours a day,” explains Richard Moxon, a professor of pediatrics with the Oxford Vaccine Group, University of Oxford. “Even brief interruptions in power could be detrimental to the proper maintenance of these vaccines.” In developed countries, plug-in refrigerators with backup generators store vaccines safely, but in developing countries, where electricity supplies can be unreliable, alternative refrigeration technologies are required. One of the most commonly used alternatives is the kerosene fridge, of which there may be over 100,000 around the world. However, these devices burn a liter of kerosene per day, which is costly and smelly, and together they may be responsible for the production of an annual 91 million kg of carbon dioxide, according to the SolarChill website. Furthermore, they cannot always be relied upon to keep vaccines cold enough. Solar-powered refrigerators are also available, but those currently on the market are expensive and require heavy lead-acid batteries to keep them running in the absence of sunlight. In addition, these batteries require maintenance, must be replaced about every three years, and must be disposed of as hazardous waste. The SolarChill fridge, however, is designed to do away with all these concerns.

## From Light to Cold

In principle, the SolarChill fridge works like any other refrigerator: a power supply drives a compressor that pushes a refrigerant around a heat exchange mechanism. In this case, however, the energy is supplied by three 60-watt solar panels. When sufficient light is captured, a DC compressor running at 3500 rpm pushes the refrigerant through the cooling system to form ice in a compartment separate from the vaccine storage unit—ice that keeps the fridge cold even after the sun goes down. Current solar-powered fridges have no such ice packs; rather, they rely on battery power to keep the fridge cool during the night. “The ice packs which are formed when the sunlight is there are the equivalent of a battery; they provide the required cooling for the vaccines when there is insufficient light,” explains Rajendra Shende, chief of the UNEP OzonAction Branch in Paris.

The cooling capacity of the ice storage unit is similar to that of a lead-acid battery, according to Per Henrik Pedersen, SolarChill project manager at the Danish Technological Institute in Taastrup. Under normal usage it can run the fridge for four to five days without being replenished by sunlight.

Even with an overcast sky the solar panels produce enough energy to keep the fridge running at full tilt. It will probably take longer to reach 2–8ºC if first powered up on a cloudy day, according to Shende, “but once it’s there it will stay at that temperature.”

The SolarChill fridge also provides environmental advantages beyond the absence of lead-acid batteries. It is the first to fully incorporate “Greenfreeze” technology, a system developed by Greenpeace in the early 1990s. The circulating refrigerant is isobutane rather than freon (a chlorofluorocarbon), and thus does not damage the ozone layer; the insulating foam in the fridge walls is blown using cyclopentane, again in place of a chlorofluorocarbon. In addition, the SolarChill unit burns no hydrocarbons and therefore produces no carbon dioxide.

## Limitations

Getting a 50-liter-capacity fridge to a place where the road may not go is a problem no matter what refrigeration technology is used. Having to transport solar panels as well makes it no easier. However, solar panels are fairly robust, says Sanford A. Klein, a professor of engineering at the University of Wisconsin–Madison. “They are packaged in a metal framework that prevents them from being twisted or bent,” he explains. “Many do have a glass surface which should be protected from sharp blows, but any reasonable type of packaging should allow them to travel.”

Klein adds that solar panels should not require maintenance. “Some types slowly degrade so that their power output decreases with time, but this is not likely an issue, as this degradation is slow,” he says. It may also be possible to reduce the size of the panels in the future. Klein explains, “There are high-efficiency solar cells that require less area to provide the same power, but they are considerably more expensive and likely not competitive since the size of the panel is not as important an issue as its cost.”

However, other researchers are developing a light-capturing technology that could eventually provide an alternative to bulky solar panels. “Quantum dot” technology involves making a solution of particle-size semiconductors that capture light, including the infrared wavelengths currently unused by solar panels, to produce electricity. Since they can be painted onto any available surface, these solutions could turn rooftops into solar panels. “Researchers are working towards ‘paint-on’ solar cells that would allow efficient solar collection for a dramatically reduced cost,” says Ted Sargent, a professor of nanotechnology at the University of Toronto. “If we succeed in making solution-processed solar cells that are [efficient and cheap], this could help the system vendors significantly reduce the cost of their offering.”

Certainly the SolarChill fridge, with its projected $2,000 price tag, does not come cheap. However, explains Shende, its zero maintenance and fuel costs should be balanced against the running costs and transport troubles of kerosene fridges. Further, the SolarChill is still less than half the price of solar-powered fridges of similar size now on the market, which cost up to $4,500, not including replacement batteries.

## Successful Field Trials

In 2004 and 2005 the SolarChill was successfully field-tested in rural health clinics in Cuba by GTZ Proklima and in Senegal and Indonesia by PATH. “After an initial observation period, health supervisors in Indonesia felt the unit was appropriate for use with vaccines, so they discontinued use of their old kerosene-powered refrigerators and used the SolarChills for storage of all vaccines,” says Carib Nelson, PATH team leader for technology solutions. “Occasional temperature fluctuations outside the required range were observed, and these led the SolarChill design team to make some minor modifications that resulted in stable temperature performance. Ministry of health officials felt the SolarChill offered a major advantage over other vaccine refrigerators due to reduced maintenance and energy costs.” Nelson adds that Indonesia has indicated interest in purchasing SolarChill once it is WHO-approved and commercially available.

WHO representative Umit Kartoglu says the organization is currently discussing the final specifications that the fridge should meet before receiving WHO approval. The developers hope that final approval will be given in the next few months. A 100-liter-capacity upright cabinet model of the fridge is also being developed for keeping food cool. These food coolers could be used in commercial settings, helping preserve milk, meat, and fruit that would otherwise quickly spoil in the tropics. The aim is that these units also be used in the domestic setting, although the current price would likely be prohibitive for most families in poorer countries. Mass production could, however, bring prices down.

SolarChill technology will not be patented; manufacturers will have open access to it in the hope that this will increase its availability to developing countries. However, in a recent meeting of the development partners in Paris, it was decided that some form of control should be maintained over which companies can manufacture SolarChill fridges. “We decided on developing some kind of a technology deployment strategy,” says Shende. “This technology [is not] intended to be patented. But at the same time we would like to . . . ensure that quality is maintained. The WHO is going to oversee the prequalification of the manufacturers, because after all, we are risking the lives of people receiving vaccinations.”

“This [technology] could provide an enormous advantage in many situations in socioeconomically deprived countries where electricity may not be available or its supply is unreliable,” concludes Moxon. Sunshine, a natural resource with which so many developing countries are blessed, may soon provide them with enough ice to overcome this problem—ice green enough for us all to benefit.

## Figures and Tables

**Figure:**
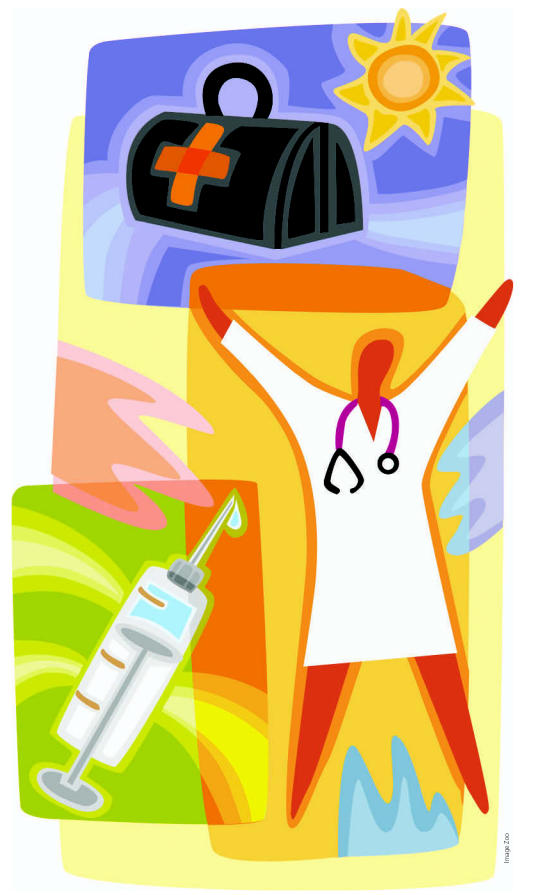


**Figure:**
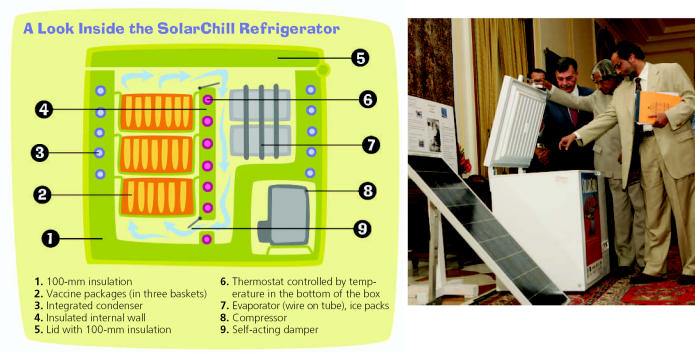
Hot technology India’s president A.P.J. Abdul Kalam views the SolarChill fridge, which should soon earn WHO approval.
